# Experiences of loneliness: a study protocol for a systematic review and thematic synthesis of qualitative literature

**DOI:** 10.1186/s13643-020-01544-x

**Published:** 2020-12-06

**Authors:** Phoebe E. McKenna-Plumley, Jenny M. Groarke, Rhiannon N. Turner, Keming Yang

**Affiliations:** 1 School of Psychology, Queen’s University Belfast, David Keir Building, 18-30 Malone Road, Belfast, BT9 5BN UK; 2 Department of Sociology, Durham University, 29-32 Old Elvet, Durham, DH1 3HN UK

**Keywords:** Loneliness, Experiences of loneliness, Systematic review, Thematic synthesis, Qualitative

## Abstract

**Background:**

Loneliness is a highly prevalent, harmful, and aversive experience which is fundamentally subjective: social isolation alone cannot account for loneliness, and people can experience loneliness even with ample social connections. A number of studies have qualitatively explored experiences of loneliness; however, the research lacks a comprehensive overview of these experiences. We present a protocol for a study that will, for the first time, systematically review and synthesise the qualitative literature on experiences of loneliness in people of all ages from the general, non-clinical population. The aim is to offer a fine-grained look at experiences of loneliness across the lifespan.

**Methods:**

We will search multiple electronic databases from their inception onwards: PsycINFO, MEDLINE, Scopus, Child Development & Adolescent Studies, Sociological Abstracts, International Bibliography of the Social Sciences, CINAHL, and the Education Resource Information Center. Sources of grey literature will also be searched. We will include empirical studies published in English including any qualitative study design (e.g. interview, focus group). Studies should focus on individuals from non-clinical populations of any age who describe experiences of loneliness. All citations, abstracts, and full-text articles will be screened by one author with a second author ensuring consistency regarding inclusion. Potential conflicts will be resolved through discussion. Thematic synthesis will be used to synthesise this literature, and study quality will be assessed using the Joanna Briggs Institute Critical Appraisal Checklist for Qualitative Research. The planned review will be reported according to the Enhancing Transparency in Reporting the Synthesis of Qualitative Research (ENTREQ) statement.

**Discussion:**

The growing body of research on loneliness predictors, outcomes, and interventions must be grounded in an understanding of the lived experience of loneliness. This systematic review and thematic synthesis will clarify how loneliness is subjectively experienced across the lifespan in the general population. This will allow for a more holistic understanding of the lived experience of loneliness which can inform clinicians, researchers, and policymakers working in this important area.

**Systematic review registration:**

PROSPERO CRD42020178105.

## Background

Loneliness has become the focus of a wealth of research in recent years. This attention is well placed given that loneliness has been designated as a significant public health issue in the UK [1] and is associated with poor physical and mental health outcomes [2–5] and an increase in risk of death similar to that of smoking [6]. In light of this, it is concerning that recent research has found that loneliness is highly prevalent across age groups, with young people (under 25 years) and older adults (over 65 years) indicating the highest levels [7, 8].

Whilst an ever-increasing body of research is situating loneliness at its centre, there is relatively little work which focuses on the lived experience of loneliness: how loneliness feels and what makes up experiences of loneliness. Phenomena that might appear to describe loneliness, such as social isolation, are distinct from the actual experience of it. Whilst loneliness is generally characterised as the distress one experiences when they perceive their social connections to be lacking in number or quality, social isolation is the objective limitation or absence of connections [9]. Social isolation does not necessarily beget loneliness, and indeed, Hawkley and Cacioppo [3] remark on how humans can perceive meaningful social relationships where none objectifiably exist, such as with God, or where reciprocity is not possible, such as with fictional characters. Whilst associations between aloneness and loneliness have been richly demonstrated [10, 11], other research has found moderate and low correlations between social isolation and loneliness [12, 13]. These findings underline the need to better understand what makes up the subjective experience of loneliness, given that it is clearly not sufficiently captured by the objective experience of being alone. Given the subjective nature of the phenomenon, qualitative methods are particularly suited to research into experiences of loneliness, as they can aim to capture the idiosyncrasies of these experiences.

A number of qualitative studies of loneliness experiences have been carried out. In perhaps the largest study of its type, Rokach [14] analysed written accounts of 526 adults’ loneliest experience, specifically asking about their thoughts, feelings, and coping strategies. This generated a model with four major elements (self-alienation, interpersonal isolation, distressed reactions, and agony) and twenty-three components such as emptiness, numbness, and missing a specific person or relationship. Although this study offered impressive scale, the vast majority of participants were between 19 and 45 years old, and as a result, the model may underestimate factors experienced across the lifespan. The findings might be usefully integrated with more recent research which qualitatively explores loneliness in other age groups (e.g. [15]). Harmonising this research by looking closely at how people describe their experiences of loneliness and working from the bottom-up to create a fine-grained view of what makes up these experiences will provide a more holistic understanding of loneliness and how it might best be defined and ameliorated.

There are a number of available definitions of loneliness offered by researchers. The widely accepted description from Perlman and Peplau [16], for example, states that loneliness is an unpleasant and distressing subjective phenomenon arising when one’s desired level of social relations differs from their actual level. However, research lacks an overarching subjective perspective, by which we mean a description of loneliness which is grounded in accounts of people’s lived experiences. This is a significant gap in the field given that loneliness is, by its nature, a subjective experience. Unlike objective phenomena like blood pressure or age, loneliness can only be definitively measured by asking a person whether they feel lonely. Weiss [17] argued that whilst available definitions of loneliness may be helpful, they do not sufficiently reflect the real phenomenon of loneliness because they define it in terms of its potential causes rather than the actual experience of being lonely. As such, studies which begin from definitions of loneliness like these may obscure the ways in which it is actually experienced and fail to capture the components and idiosyncrasies of these experiences.

A recent systematic review report [18] has explored the conceptualisations of loneliness employed in qualitative research, finding that loneliness tended to be defined as social, emotional, or existential types. However, the review covered only studies of adults (16 years and up), including heterogenous clinical populations (e.g. people receiving cancer treatment, people living with specific mental health conditions, and people on long-term sick leave), and placed central importance on the concepts, models, theories, and frameworks of loneliness utilised in research. Studies which did not employ an identified concept, model, framework, or theory of loneliness were excluded. Moreover, rather than synthesising how people describe their loneliness, the authors aimed to assess how research conceptualises loneliness across the adult life course. This leaves a gap with respect to how research participants specifically describe their lived experiences of loneliness, rather than how researchers might conceptualise it. Achterberg and colleagues [19] recently conducted a meta-synthesis of qualitative studies on experiences of loneliness in young people with depression. As the findings are specific to experiences in this population, they may not reflect those of wider age groups or individuals who do not have depression. Kitzmüller and colleagues [20] used meta-ethnography to synthesise studies regarding experiences and ways of dealing with loneliness in older adults (60 years and older). However, they synthesised only articles from health care disciplines published in scientific journals from 2001 to 2016 and included studies on clinical populations, such as older women with multiple chronic conditions. Moreover, there has been an increase in research output regarding loneliness in recent years, and relevant studies may have been published since this review was conducted (e.g. [15]). To the authors’ knowledge, the systematic review report on conceptual frameworks used in loneliness research [18], the meta-synthesis of loneliness in young people with depression [19], and the meta-ethnography of older adults’ loneliness [20] are the only such systematic reviews of qualitative literature regarding experiences of loneliness to date. The current systematic review will instead take a bottom-up approach which focuses on non-clinical populations of all ages to synthesise findings on participants’ experiences of loneliness, rather than the conceptualisations that might be imposed by study authors. This will fill a gap in the literature by synthesising the qualitative evidence focusing on experiences of loneliness across the lifespan. This inductive synthesis of the available subjective descriptions of loneliness will offer a nuanced view of loneliness experiences. It is imperative for research and practice that we deepen the current understanding of these experiences to inform how we approach describing, researching, and attempting to ameliorate loneliness.

### Aims

The proposed research aims to offer a holistic view of the experience of loneliness across the lifespan through a systematic review and thematic synthesis of the qualitative literature focusing on these experiences. To address this aim, there is one central research question: How do people describe their experiences of loneliness?

This research question concerns aspects of loneliness which participants discuss when describing their lived experiences. Whilst we expect that this would concern emotional, social, and cognitive components of the experience, we understand that these findings may also come to reflect perceived causes or effects of loneliness.

This review will also consider the age groups that have been studied and how experiences of loneliness might vary across the different age groups examined in this literature. Loneliness research is often weighted towards investigations of older adults, despite the fact that the prevalence of loneliness is high across the lifespan; recent UK research found a prevalence of 40% in 16- to 24-year-olds and 27% in people over 75 [7]. This review will also shed light on the age groups that have been included in qualitative research on loneliness experiences. In doing so, this research may identify age groups which have been understudied and may be underrepresented in this field of research, potentially pointing to life stages where experiences of loneliness might be usefully explored in more detail in the future.

Furthermore, given the relatively small number of qualitative studies into the experience of loneliness compared with quantitative research in this area, this review will also consider the reasons that study authors may offer for the relative shortage of qualitative work. This is an important point given that the review will inherently be constrained by the number of studies that exist and the focus that has primarily been given to quantitative loneliness research thus far.

## Methods

### Protocol registration and reporting

The review protocol has been registered within the International Prospective Register of Systematic Reviews (PROSPERO) database from the University of York (registration number: CRD42020178105). This review protocol is being reported in accordance with the reporting guidance provided in the Preferred Reporting Items for Systematic Reviews and Meta-Analyses Protocols (PRISMA-P) statement [21] (see checklist in Additional file 1). The proposed systematic review will be reported in accordance with the reporting guidance provided in the Enhancing Transparency in Reporting the Synthesis of Qualitative Research (ENTREQ) statement [22]. The Preferred Reporting Items for Systematic Reviews and Meta-Analyses (PRISMA) statement [23] will inform the process of completing and reporting this planned review.

### Eligibility criteria

Due to its suitability for qualitative evidence synthesis, the SPIDER tool [24] was used to assist in defining the research question and eligibility criteria in line with the following criteria: Sample, Phenomenon of Interest, Design, Evaluation, Research type (see Table 1 for details of these criteria). The exclusion criteria are as follows:

1. Studies not meeting the inclusion criteria described in Table 1
2. Studies not published in English
3. Studies with no qualitative component
4. Studies of clinical populations
5. Studies which report solely on objective phenomena such as social isolation rather than the subjectively perceived experience of loneliness
6. Studies in which the primary focus or one of the primary focuses is not experiences of loneliness

**Table 1:** Application of the SPIDER criteria to the research question and eligibility criteria of the current study

SPIDER criterion	The current study
Sample	Individuals of any age who describe experiences of loneliness, not including specific clinical populations.
Phenomenon of interest	Loneliness.
Design	Any qualitative research design, e.g. interview or focus group. Mixed-methods designs which include qualitative methods will be included if the qualitative findings are reported separately.
Evaluation	Descriptions of experiences of loneliness.
Research type	Primary qualitative research.

Papers will be deemed to focus sufficiently on experiences of loneliness if studying these experiences is a key aspect of the work rather than simply a part of the output. Accordingly, studies will only be included if authors state a relevant aim, objective, or research question related to investigating experiences of loneliness (i.e. to study experiences of loneliness) or if loneliness experiences are clearly explored and described (e.g. relevant questions are present in an appended interview guide). At the title and abstract screening stage, at least one relevant sentence or information that indicates likely relevance must be present for inclusion. The decision to exclude articles which do not primarily or equally focus on these experiences was made in order to gather meaningful data about loneliness experiences specifically and to capture experiences identified as loneliness by participants as much as possible, rather than related phenomena which may be grouped and labelled retrospectively as loneliness by researchers.

### Information sources and search strategy

The primary source of literature will be a structured search of multiple electronic databases (from inception onwards): PsycINFO, MEDLINE, Scopus, Child Development & Adolescent Studies, Sociological Abstracts, International Bibliography of the Social Sciences (IBSS), CINAHL, and the Education Resource Information Center (ERIC). The secondary source of potentially relevant material will be a search of the grey or difficult-to-locate literature using Google Scholar. In line with the guidance from Haddaway and colleagues [25] on using Google Scholar for systematic review, a title-only search using the same search terms will be conducted and the first 1000 results will be screened for eligibility. These searches will be supplemented with hand-searching in reference lists, such that the titles of all articles cited within eligible studies will be checked. When eligibility is unclear from the title, abstracts and full-texts will be checked until eligibility or ineligibility can be ascertained. This process will be repeated with any articles that are found to be eligible at this stage until no new eligible articles are found. Systematic reviews on similar topics will also be searched for potentially eligible studies. Grey literature will be located through searches of Google Scholar, opengrey.eu, ProQuest Dissertations and Theses, and websites of specific loneliness organisations such as the Campaign to End Loneliness, managed in collaboration with an information specialist. Efforts will be made to contact authors of completed, ongoing, and in-press studies for information regarding additional studies or relevant material.

The search strategy for our primary database (MEDLINE) was developed in collaboration with an information specialist. In collaboration with a specialist, the strategy will be translated for all of the databases. The search strategy has been peer reviewed using the Peer Review of Electronic Search Strategies (PRESS) checklist [26]. Strategies will utilise keywords for loneliness and qualitative studies. A draft search strategy for MEDLINE is provided in Additional file 2. Qualitative search terms were supplemented with relevant and useful subject headings and free-text terms from the Pearl Harvesting Search Framework synonym ring for qualitative research [27]. The inclusion of search terms related to social isolation specifically and related terms (e.g. “social engagement”) was considered and tested extensively through scoping searches and discussion with an information specialist. Adding these terms (and others such as “Patient Isolation” and “Quarantine”) did not appear to add unique papers that would be included above and beyond subject heading and free-text searching for “Loneliness”. Given the aim to include studies focused on experiences of loneliness specifically, this search strategy was deemed most appropriate. A similar strategy has been employed in other recent systematic review work focusing on loneliness (e.g. [28, 29]). Moreover, test searches employing the search strategy retrieved all of seven informally identified likely eligible articles indexed in Scopus, indicating good sensitivity of the strategy. A free-text search to capture “perceived social isolation” was included as this specific term is used by some authors as a direct synonym for loneliness. The completed PRESS checklist is provided in Additional file 3.

### Data collection and analysis

#### Study selection

Firstly, the main review author (PMP) will perform the database search and hand-searching and will screen all titles to remove studies which are clearly not relevant. PMP will also undertake abstract screening to exclude any which are found to be irrelevant or inapplicable to the inclusion criteria. A second author (JG) will independently screen 50% of the titles and abstracts. Finally, full-text versions of the remaining articles will be read by PMP to assess whether they are suitable for inclusion in the final review. JG will independently review 50% of these full texts. In cases of disagreement, the two reviewers will discuss the study to reach a decision about inclusion or exclusion. In case agreement cannot be reached after discussion between the two reviewers, a third reviewer will be invited to reconcile their disagreement and make a final decision. The reason for the exclusion at the full-text stage will be recorded. After this screening process, the remaining articles will be included in the review following data extraction, quality appraisal, and analysis. The PRISMA statement will be followed to create a flowchart of the number of studies included and excluded at each stage of this process.

#### Data management

The articles to be screened will be managed in EndNoteX9, with subsequent EndNote databases used to manage each stage of the screening process.

#### Data extraction

Data will be extracted from the studies by PMP using a purpose-designed and piloted Microsoft Excel form. Information on author, publication year, geographic location of study, methodological approach, method, population, participant demographics, and main findings will be extracted to understand the basis of each study. JG will check this extracted data for accuracy.

For the thematic synthesis, in line with Thomas and Harden [30], all text labelled as “results” or “findings” will be extracted and entered into the NVivo software for analysis. This will be done because many factors, including varied reporting styles and misrepresentation of data as findings, can make it difficult to identify the findings in qualitative research [31]; accordingly, a wide-ranging approach will be used to capture as much relevant data as possible from each included article. The aim is to extract all data in which experiences of loneliness are described.

#### Quality appraisal

Quality of the included articles will be assessed using the Joanna Briggs Institute (JBI) Critical Appraisal Checklist for Qualitative Research [32]. This quality will be considered during the development of the data synthesis. Different authors hold different viewpoints about inclusion versus exclusion of low-quality studies. However, given that they may still add important, authentic accounts of phenomena that have simply been reported inadequately [33], it is common to include lower-quality studies and consider quality during the synthesis process rather than excluding on the basis of it. Accordingly, this approach will be used in the present research.

#### Data synthesis

There are various accepted approaches to reviewing and synthesising qualitative research, including meta-ethnography [34], meta-synthesis [35], and narrative synthesis [36]. The current systematic review will utilise thematic synthesis as a methodology to create an overarching understanding of the experiences of loneliness described across studies. In thematic synthesis, descriptive themes which remain close to the primary studies are developed. Next, a new stage of analytical theme development is undertaken wherein the reviewer “goes beyond” the interpretations of the primary studies and develops higher-order constructs or explanations based on these descriptive themes [30]. The process of thematic synthesis for reviewing is similar to that of grounded theory for primary data, in that a translation and interpretative account of the phenomena of interest is produced. Thematic synthesis has been used to synthesise research on the experience of fatigue in neurological patients with multiple sclerosis [37], children’s experiences of living with juvenile idiopathic arthritis [38], and parents’ experiences of parenting a child with chronic illness [39]. This use of thematic synthesis to consider subjective experiences (rather than, for example, attitudes or motivations) melds well with the present research, which also sets its focus on a subjective experience.

As well as its successful application in similar systematic reviews, thematic synthesis was selected based on its appropriateness to the research question, time frame, resources, expertise, purpose, and potential type of data in line with the RETREAT framework for selecting an approach to qualitative evidence synthesis [40]. The RETREAT framework considers thematic synthesis to be appropriate for relatively rapid approaches which can be sustained by researchers with primary qualitative experience, unlike approaches such as meta-ethnography in which a researcher with specific familiarity with the method is needed. This is appropriate to the project time frame and background of this research team. The Joanna Briggs Institute Reviewer’s Manual [41] also notes that thematic synthesis is useful when considering shared elements across studies which are otherwise heterogenous, which is likely to be the case in this review given that the common factor (experiences of loneliness) may be present across studies with otherwise diverse populations and methodologies.

Guidance from Thomas and Harden [30] will be followed to synthesise the data. Firstly, the extracted text will be inductively coded line-by-line according to content and meaning. This inductive creation of codes should allow the content and meaning of each sentence to be captured. Multiple codes may be applied to the same sentence, and codes may be “free” or structured in a tree formation at this stage. Before moving forward, all text referred to by each code will be rechecked to ensure consistency in what is considered a single code or whether more levels of coding are required.

After this stage, similarities and differences between the codes will be examined, and they will begin to be organised into a hierarchy of groups of codes. New codes will be applied to these groups to describe their overall meaning. This will create a tree structure of descriptive themes which should not deviate largely from the original study findings; rather, findings will have been integrated into an organised whole. At this stage, the synthesis should remain close to the findings of the included studies.

At the final stage of analysis, higher-order analytical themes may be inferred from the descriptive themes which will offer a theoretical structure for experiences of loneliness. This inferential process will be carried out through collaboration between the research team (primarily PMP and JG).

#### Sensitivity analysis

After the synthesis is complete, a sensitivity analysis will be undertaken in which any low-quality studies (as identified through the JBI checklist) are excluded from the analysis to assess whether the synthesis is altered when these studies are removed, in terms of any themes being lost entirely or becoming less rich or thick [42]. Sensitivity analysis will also be used to assess whether any age group is entirely responsible for a given theme. In this way, the robustness of the synthesis can be appraised and the individual findings can remain grounded in their context whilst also extending into a broader understanding of the experiences of loneliness.

#### Risk of bias in individual studies

Risk of bias in individual studies will be taken into account through utilisation of the JBI checklist, which includes ten questions to assess whether a study is adequately conceptualised and reported [32]. PMP will use the checklist to assess the quality of each study. Whilst all eligible studies will be included in the synthesis (as described in the “Quality appraisal” section), any lower-quality studies will be excluded during post-synthesis sensitivity analysis in order to assess whether their inclusion has affected the synthesis in any way as suggested by Carroll and Booth [43].

#### Confidence in cumulative evidence

The Grading of Recommendations, Assessment, Development and Evaluation – Confidence in the Evidence from Reviews of Qualitative Research (GRADE-CERQual) approach [44, 45] will be used to assess how much confidence can be placed in the findings of this qualitative evidence synthesis. This will allow a transparent, systematic appraisal of confidence in the findings for researchers, clinicians, and other decision-makers who may utilise the evidence from the planned systematic review. GRADE-CERQual involves assessment in four domains: (1) methodological limitations, (2) coherence, (3) adequacy of data, and (4) relevance. There is also an overall rating of confidence: high, moderate, low, or very low. These findings will be displayed in a Summary of Qualitative Findings table including a summary of each finding, confidence in that finding, and an explanation for the rating. Assessments for each finding will be made through discussion between PMP and JG.

## Discussion

The proposed systematic review will contribute to our knowledge of loneliness by clarifying how it is subjectively experienced across the lifespan. Synthesising the qualitative literature focusing on experiences of loneliness in the general population will offer a fine-grained, subjectively derived understanding of the components of this phenomenon which closely reflects the original descriptions provided by those who have experienced it. By including non-clinical populations of all ages, this research will provide an essential view of loneliness experiences across different life stages. This can be used to inform future research into correlates, consequences, and interventions for loneliness. The use of thematic synthesis will enable us to remain close to the data, offering an account which might also be useful for policy and practice in this area.

There are a number of limitations to the planned research. Primarily, this review will be unable to capture aspects of loneliness experiences which have not been described in the qualitative literature, for example, due to the sensitivity of the topic, given that loneliness can be stigmatising, or aspects that are specific to a given unstudied population. Moreover, by focusing on lifespan non-clinical research, we aim to offer a general synthesis which can in future be informed by insights from clinical groups, rather than subsuming and potentially obscuring the aspects of loneliness which might be unique to them. Whilst primary empirical studies are not themselves extensive sources, with books in particular often offering rich descriptions of loneliness (see, e.g. [11, 46]), this research will focus on primary empirical studies of subjective descriptions to offer a manageable level of scope and rigour. As with any systematic review, some studies may also be missing information which would inform the synthesis. Quality appraisal and sensitivity analysis will aim to capture and potentially control for this issue, but it will ultimately be difficult to ascertain how missing information might affect the synthesis.

By providing a thorough overview of how loneliness is experienced, we expect that the findings from the planned review will be informative and useful for researchers, policymakers, and clinicians who work with and for people experiencing loneliness, as well as for these individuals themselves, to better understand this important, prevalent, and often misunderstood phenomenon. Mansfield et al. [18] have offered an illuminating systematic review covering the conceptual frameworks and models of loneliness included in the existing evidence base (i.e. social, emotional, and existential loneliness). This review will build upon this work by including research with children and adolescents and taking a bottom-up approach similar to grounded theory where the synthesis will remain close to the participants’ subjective descriptions of loneliness experiences within the included studies, rather than reflecting pre-existing themes in the evidence base. As such, this systematic review will offer specific insights into lifespan experiences of loneliness. This synthesis of lived experiences will shed light on the nuances of loneliness which existing definitions and typologies might overlook. It will offer an experience-focused overview of loneliness for people studying and developing measures of this phenomenon. In focusing on qualitative work, the planned review may also identify processes relevant to loneliness which are not expressed by statistical models. In this way, it may also provide a starting point for more nuanced qualitative work with specific populations and circumstances to ascertain components which may be characteristic of certain experiences.

## Supplementary Information

**Additional file 1.** PRISMA-P 2015 Checklist.

**Additional file 2.** Search executed in MEDLINE ALL (OVID) 16/11/2020.

**Additional file 3.** PRESS Guideline — Search Submission & Peer Review Assessment.

## Data Availability

Not applicable.

## Abbreviations

ENTREQ: Enhancing Transparency in Reporting the Synthesis of Qualitative Research
ERIC: Education Resource Information Center
GRADE-CERQual: Grading of Recommendations, Assessment, Development and Evaluation – Confidence in the Evidence from Reviews of Qualitative Research
IBSS: International Bibliography of the Social Sciences
JBI: Joanna Briggs Institute
JG: Jenny Groarke
KY: Keming Yang
PMP: Phoebe McKenna-Plumley
PRISMA: Preferred Reporting Items for Systematic Reviews and Meta-Analyses
PRISMA-P: Preferred Reporting Items for Systematic Reviews and Meta-Analyses – Protocol
PROSPERO: International Prospective Register of Systematic Reviews
RETREAT: Review question – Epistemology – Time/Timescale – Resources – Expertise – Audience and purpose – Type of data
RT: Rhiannon Turner
SPIDER: Sample, Phenomenon of Interest, Design, Evaluation, Research type
