# Supplementary material for: Experiences of loneliness: a study protocol for a systematic review and thematic synthesis of qualitative literature
Source: Syst Rev. 2020 Dec 6;9:284. doi: 10.1186/s13643-020-01544-x (PMC7720528; doi:10.1186/s13643-020-01544-x)
Supplement: Supplementary file 2 — Additional file 2. Search executed in MEDLINE ALL (OVID) 16/11/2020. [file 13643_2020_1544_MOESM2_ESM.docx]

Search executed in MEDLINE ALL (OVID) 16/11/2020:

| **#** | **Searches** | **Results** |
| --- | --- | --- |
| 1 | exp Loneliness/ | 3891 |
| 2 | lonel*.ti,ab,kw. | 7794 |
| 3 | ("social* isolat*") ADJ6 (perce*).ti,ab,kw. | 273 |
| 4 | **combine 1, 2, 3 with OR** | 8900 |
| 5 | exp Qualitative Research/ | 58215 |
| 6 | qualitative*.ti,ab,kw. | 286542 |
| 7 | "mixed method*".ti,ab,kw. | 23802 |
| 8 | "mixed-method*".ti,ab,kw. | 23802 |
| 9 | exp Interview/ | 29380 |
| 10 | interview*.ti,ab,kw. | 366409 |
| 11 | exp Focus Groups/ | 30717 |
| 12 | "focus group*".ti,ab,kw. | 48412 |
| 13 | "thematic analysis".ti,ab,kw. | 20930 |
| 14 | exp Personal Narrative/ | 9003 |
| 15 | "narrative analysis".ti,ab,kw. | 1275 |
| 16 | "narrative approach".ti,ab,kw. | 797 |
| 17 | exp Grounded Theory/ | 1775 |
| 18 | "grounded theory".ti,ab,kw. | 11938 |
| 19 | "phenomenological analysis".ti,ab,kw. | 2450 |
| 20 | "phenomenological approach".ti,ab,kw. | 2449 |
| 21 | ethnograph*.ti,ab,kw. | 11236 |
| 22 | "discourse analysis".ti,ab,kw. | 1908 |
| 23 | "content analysis".ti,ab,kw. | 29550 |
| 24 | "lived experience".ti,ab,kw. | 4187 |
| 25 | "group discussion".ti,ab,kw. | 3327 |
| 26 | "case stud*".ti,ab,kw. | 100271 |
| 27 | "audio record*".ti,ab,kw. | 6803 |
| 28 | "audiorecord*".ti,ab,kw. | 369 |
| 29 | lifeworld*.ti,ab,kw. | 505 |
| 30 | "life world*".ti,ab,kw. | 491 |
| 31 | "life-world*".ti,ab,kw. | 491 |
| 32 | "constant comparative".ti,ab,kw. | 3207 |
| 33 | "constant comparison".ti,ab,kw. | 1588 |
| 34 | "biographical method*".ti,ab,kw. | 27 |
| 35 | "open-ended".ti,ab,kw. | 14755 |
| 36 | "open ended".ti,ab,kw. | 14755 |
| 37 | **combine 5, 6, 7, 8, 9, 10, 11, 12, 13, 14, 15, 16, 17, 18, 19, 20, 21, 22, 23, 24, 25, 26, 27, 28, 29, 30, 31, 32, 33, 34, 35, 36 with OR** | 735269 |
| 38 | **4 AND 37** | 1988 |
